# Correction: Yao, Y., et al. Activation of Slit2/Robo1 Signaling Promotes Tumor Metastasis in Colorectal Carcinoma through Activation of the TGF-β/Smads Pathway. *Cells* 2019, *8*, 635

**DOI:** 10.3390/cells9081918

**Published:** 2020-08-18

**Authors:** Yuying Yao, Zijun Zhou, Liuyou Li, Junchen Li, Lixun Huang, Jiangchao Li, Cuiling Qi, Lingyun Zheng, Lijing Wang, Qian-Qian Zhang

**Affiliations:** Vascular Biology Research Institute, School of Life Sciences and Biopharmaceutics, Guangdong Pharmaceutical University, Guangzhou 510006, China; yaoyuying93@163.com (Y.Y.); zhouzijun72@163.com (Z.Z.); 18028014631@163.com (L.L.); lijunchen96@163.com (J.L.); gdpuhlx@163.com (L.H.); lijiangchao1234@163.com (J.L.); qicuiling12345@163.com (C.Q.); freeflyzly@hotmail.com (L.Z.)

The author wishes to make the following correction to this paper [1]. Due to the authors having made an error, the Smad2/3 IHC figure of the R5 group in Figure 4E and figures in Supplementary Figure S3D, Figure 4 and Supplementary Figure S3 need to be corrected. Please replace 
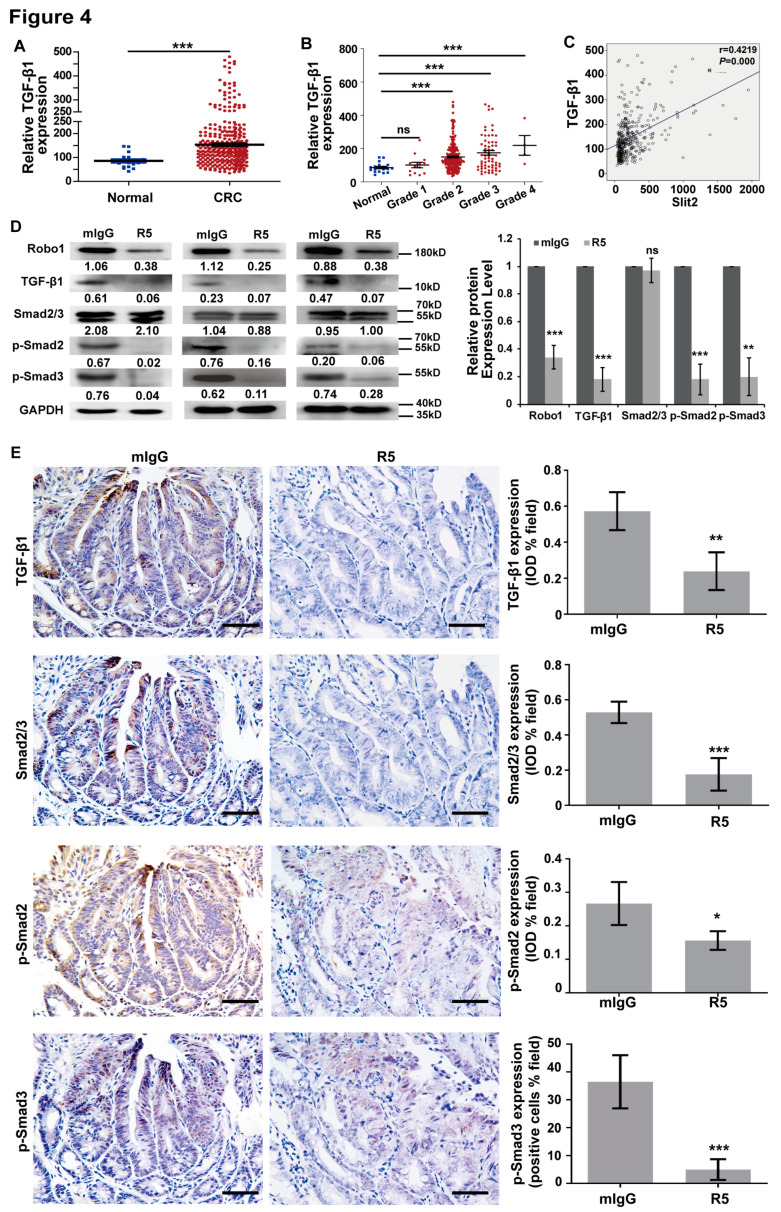
 with 
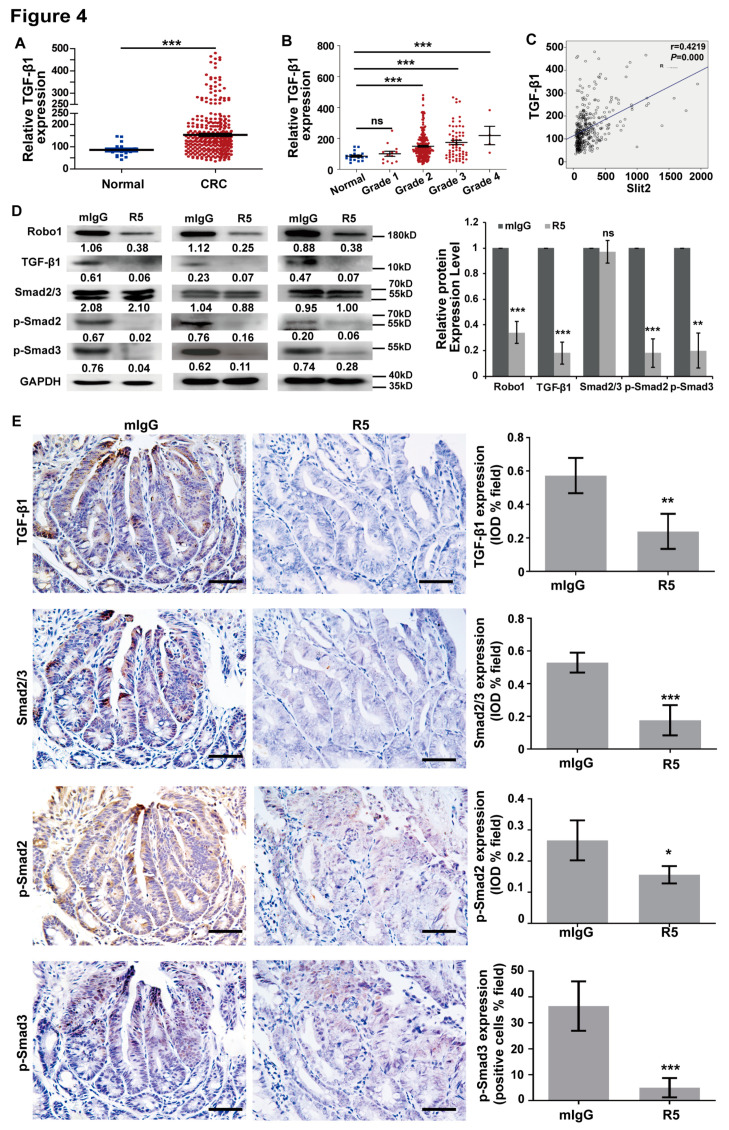
 and 
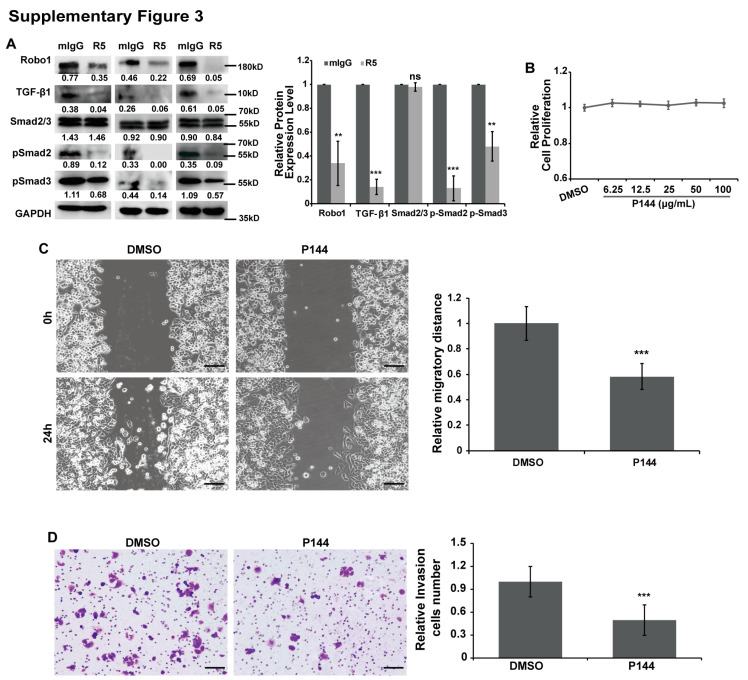
 with 
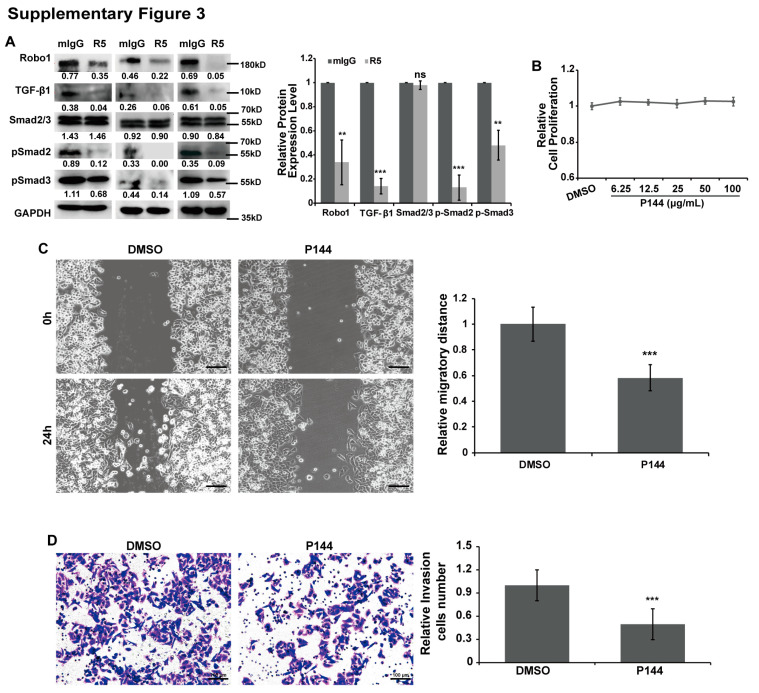


The authors would like to apologize for any inconvenience caused to the readers by these changes. These corrections do not affect the study’s results or conclusions.
