# Supplementary material for: Activation of Slit2/Robo1 Signaling Promotes Tumor Metastasis in Colorectal Carcinoma through Activation of the TGF-β/Smads Pathway
Source: Cells. 2019 Jun 25;8(6):635. doi: 10.3390/cells8060635 (PMC6628122; doi:10.3390/cells8060635)
Supplement: Supplementary file 1 [file cells-08-00635-s001.pdf]

Supplementary Figure Legends

Supplementary Figure 1

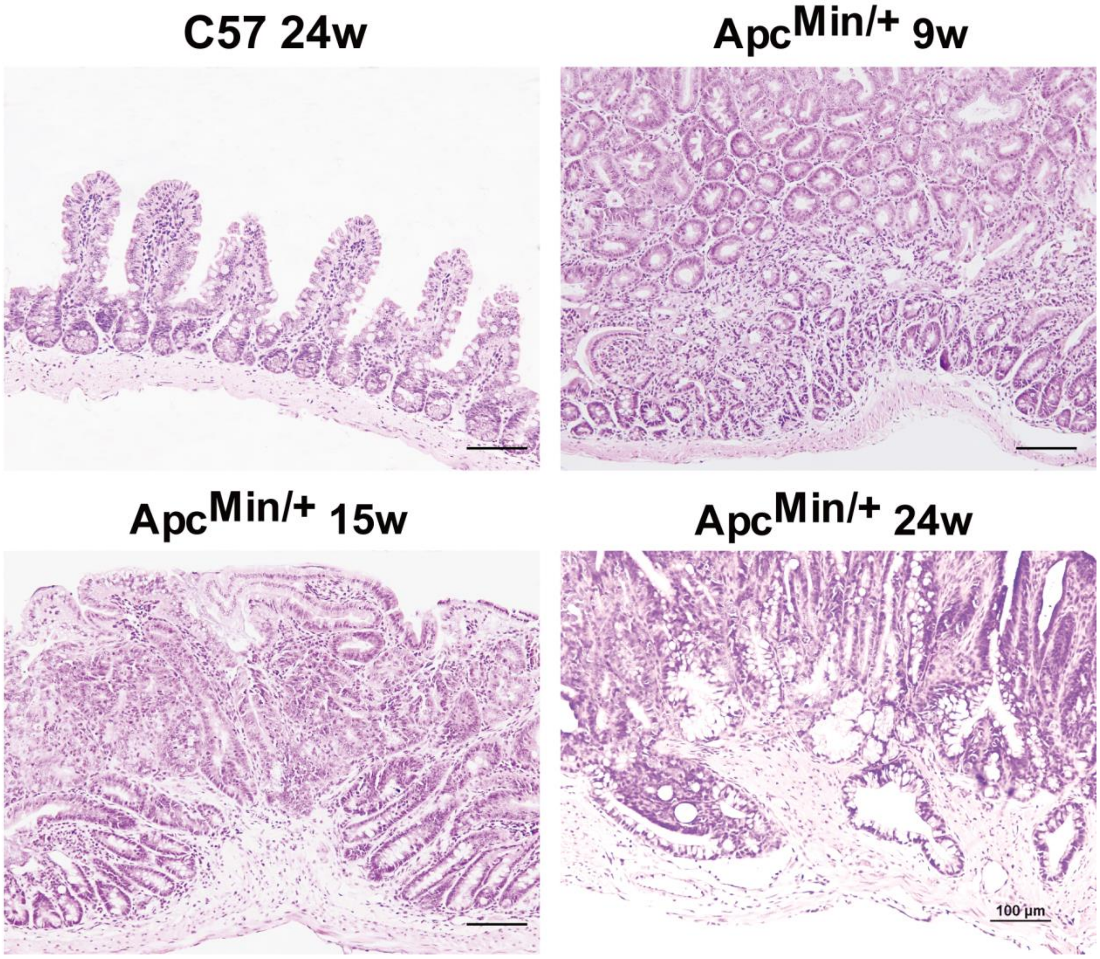

Supplementary Figure 1. The pathological stages of Apc$^{Min/+}$ mice. The pathological process in Apc$^{Min/+}$ mice was accessed under a microscope through H&E staining**.** Hyperplasia of the small intestine glands can be seen in most of the small intestines of 9 week old Apc$^{Min/+}$ mice. In 15 week old Apc$^{Min/+}$ mice, the basal lamina was intact and the epithelial cells were well differentiated. In addition, Adenoma can be seen in most of the small intestines of 15 week old Apc$^{Min/+}$ mice. Adenocarcinoma can be seen in most of the small intestines of 24 week old Apc$^{Min/+}$ mice, and some tumor cells had broken through the basement. Scale Bar: 100 µm.

Supplementary Figure 2

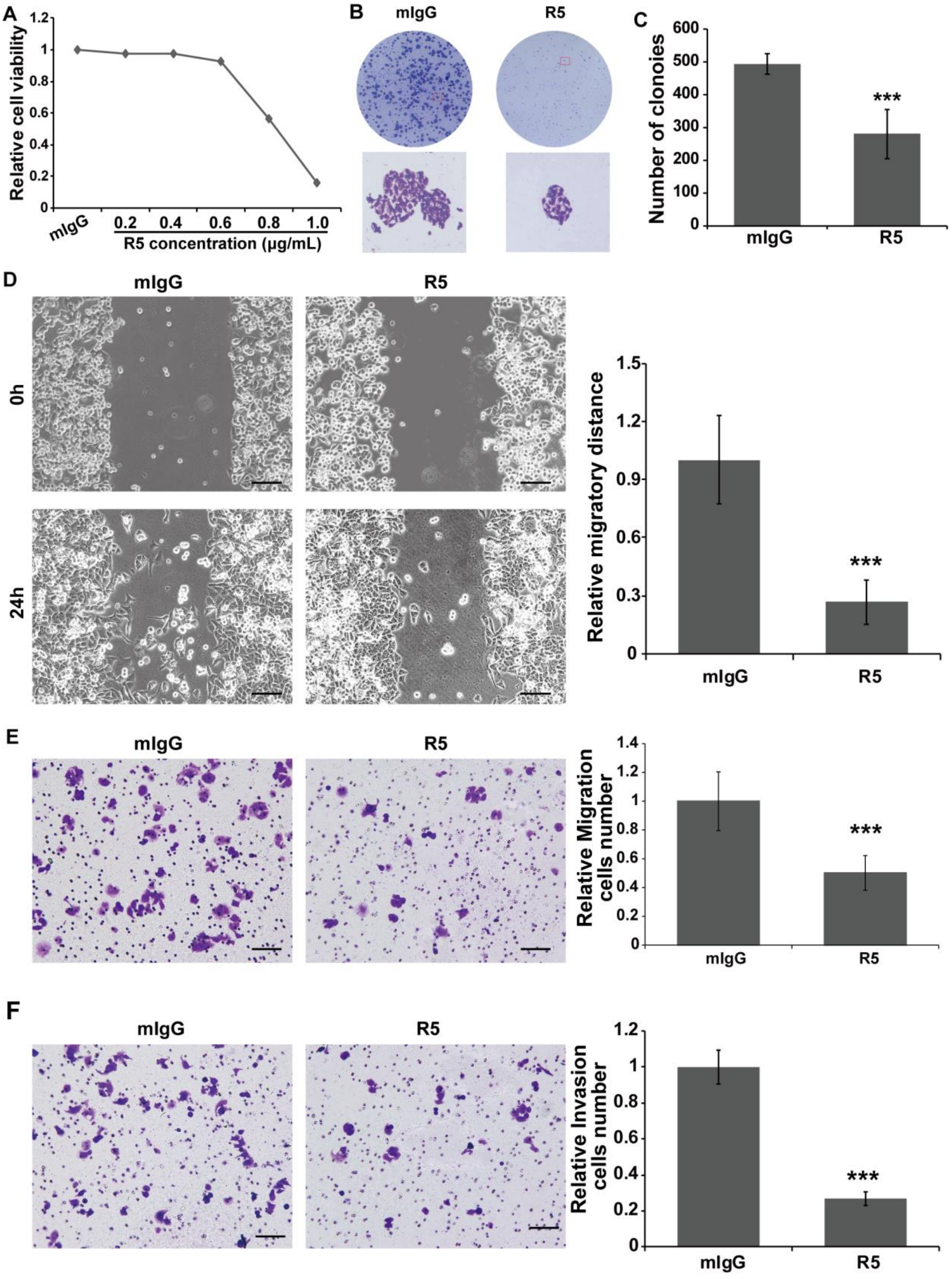

Supplementary Figure 2. Blocking of Slit2/Robo1 signaling inhibits cell proliferation, migration, chemotatic and invasion of SW480 cells *in vitro*. (**A**) SW480 cells were treated with indicated concentration of R5 for 48 h. The cell viability was measured using CCK8 assay. R5 inhibits cell viability in a dose-dependent manner,

and the IC_{50} is ～0.8 μg/mL in SW480 cells. (**B**) SW480 cells (1000 cell per well) were treated with 0.8 μg/mL of mIgG or R5. The size of colonies wad smaller in R5-treated wells compared with mIgG-treated wells. (**C**) The total number of colonies in R5-treated and mIgG-treated wells of SW480 cells were counted, and fewer colonies can be seen in R5-treated wells compared with mIgG-treated wells. The results are representative of three independent experiments. (**D**) The inhibition effect of blocking of Slit2/Robo1 signaling on cell migration was detected using a wound healing assay. SW480 cells were treated with mIgG or R5 at 0.8 μg/mL, and the relative cell migration distance compared with 0 h was detected and statistic at 24 hours later. R5 significantly inhibited cell migration compared with mIgG in SW480 cells. (**E**) The chemotatic ability of SW480 cells that treated with R5 or mIgG was detected using tanswell migration assay. The cells were treated with mIgG or R5 (0.8 μg/mL) and the cells migrated to the lower membrane of chamber was detected at 16 h later. The number of migrated cells were decreased by R5 compared with mIgG treated group. (**F**) A Matrigel invasion assay was used to detect the inhibition effect of R5 on cell invasion. SW480 cells were added into the upper chambers and treated with mIgG or R5 at 0.8 μg/mL, and the invasion of the cells to the bottom of the chambers was measured 20 h later. Specific blocking of Slit2/Robo1 signaling using R5 significantly suppresses cell invasion in SW480 cells compared with mIgG. ***P< 0.001. Scale Bars: 100 μm.

Supplementary Figure 3

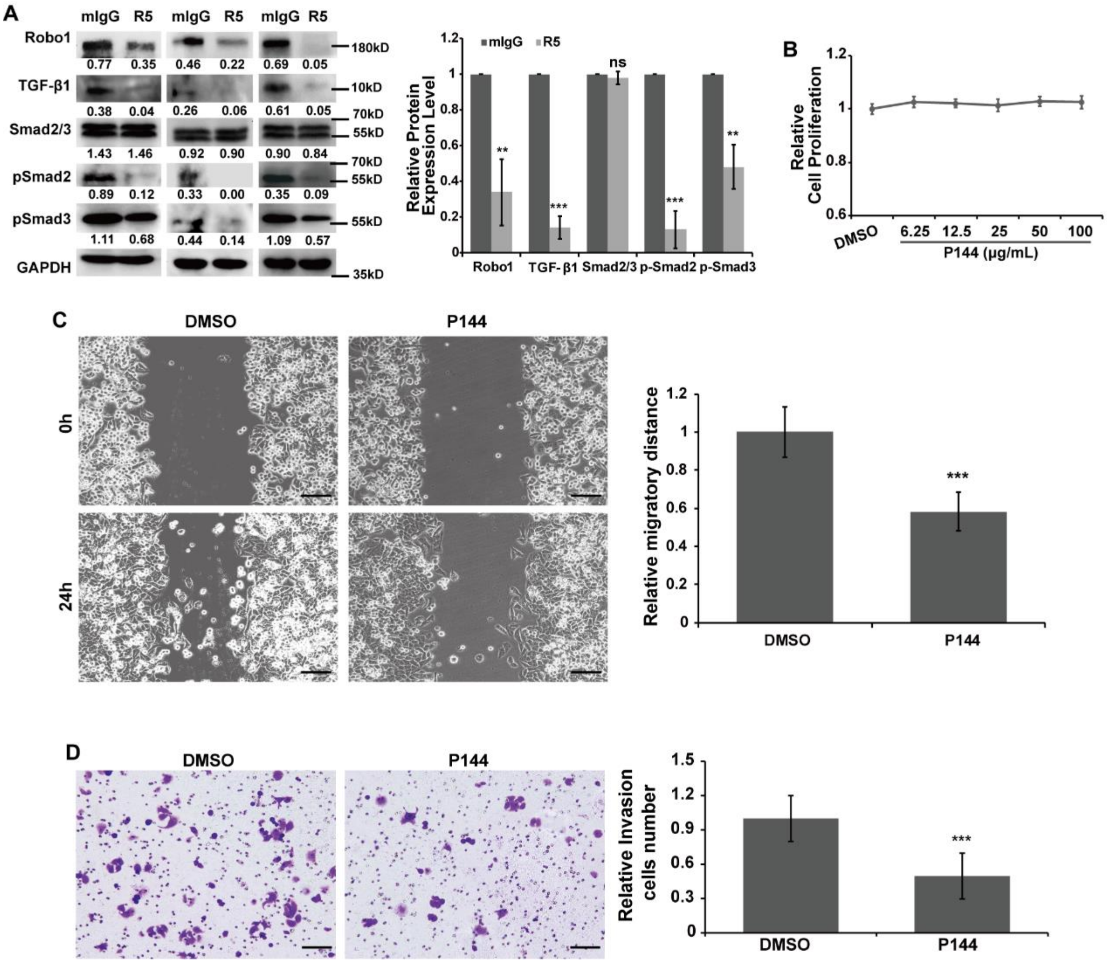

Supplementary Figure 3. TGF-β1/Smad2/3 pathway is correlated with Slit2/Robo1 signaling inhibits cell migration and invasion in SW480 cells. (A) Blocking of Slit2/Robo1 signaling using R5 significantly inhibit the expression of TGF-β1 and phosphorylated Smad2/Smad3 in SW480 cells. (B) Cell proliferation was not affected by P144, a TGF-β1 inhibitor, in SW480 cells at 48 h post-treatment. The cell migration and invasion ability of SW480 cells were measured using wound healing assay and Matrigel invasion assay. The cell migration (C) and invasion (D) ability was significantly inhibited by P144 compared with the cells were treated with DMSO in SW480 cells. ns: no significantly difference, **: $P < 0.05$, ***: $P < 0.001$. The protein bands were quantified densitometrical and normalized to GAPDH expression. Scale Bars: 100 μm.

Supplementary Figure 4

A. The uncut gels of western blot for three replicates in figure 4D.

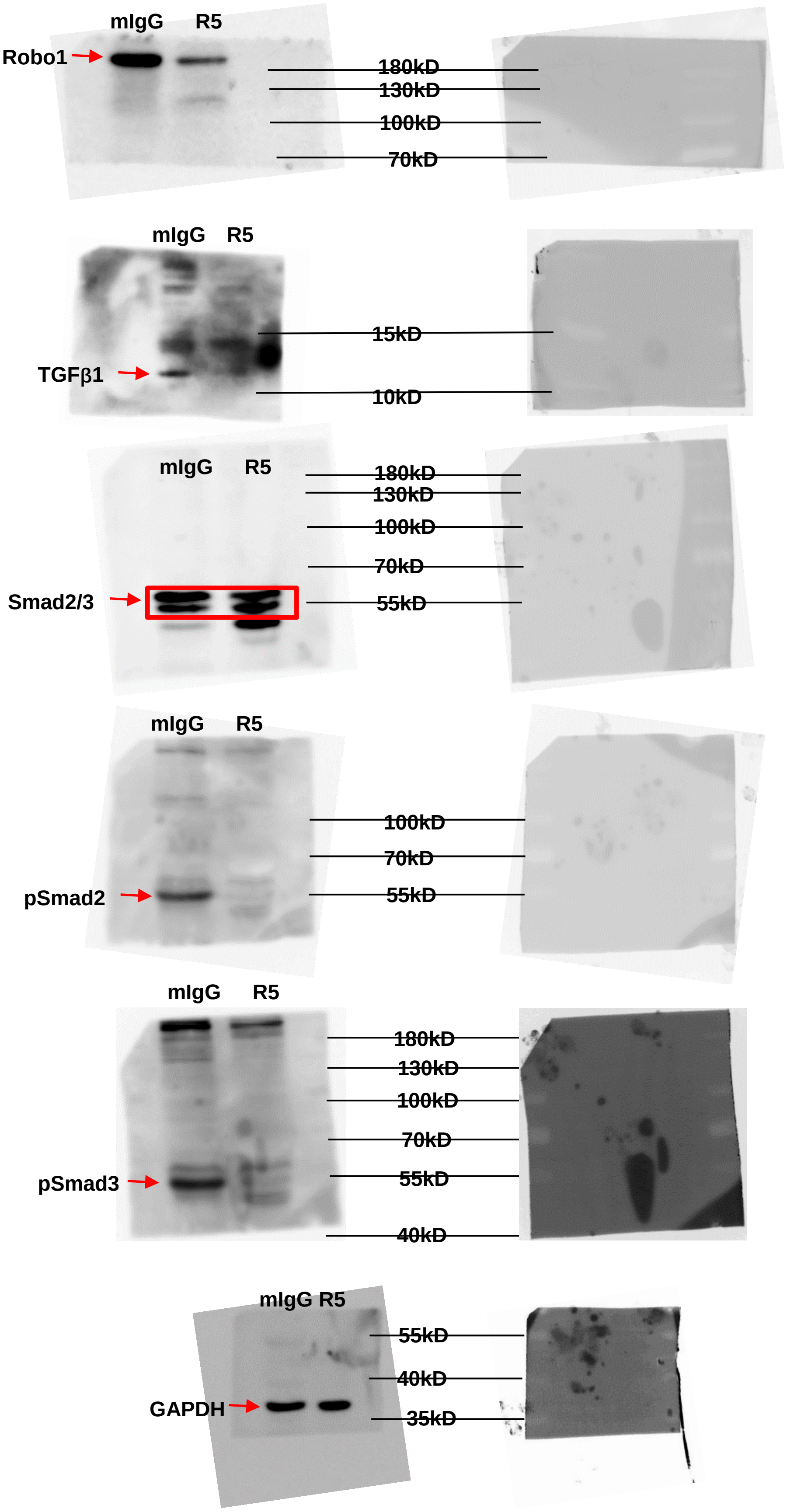

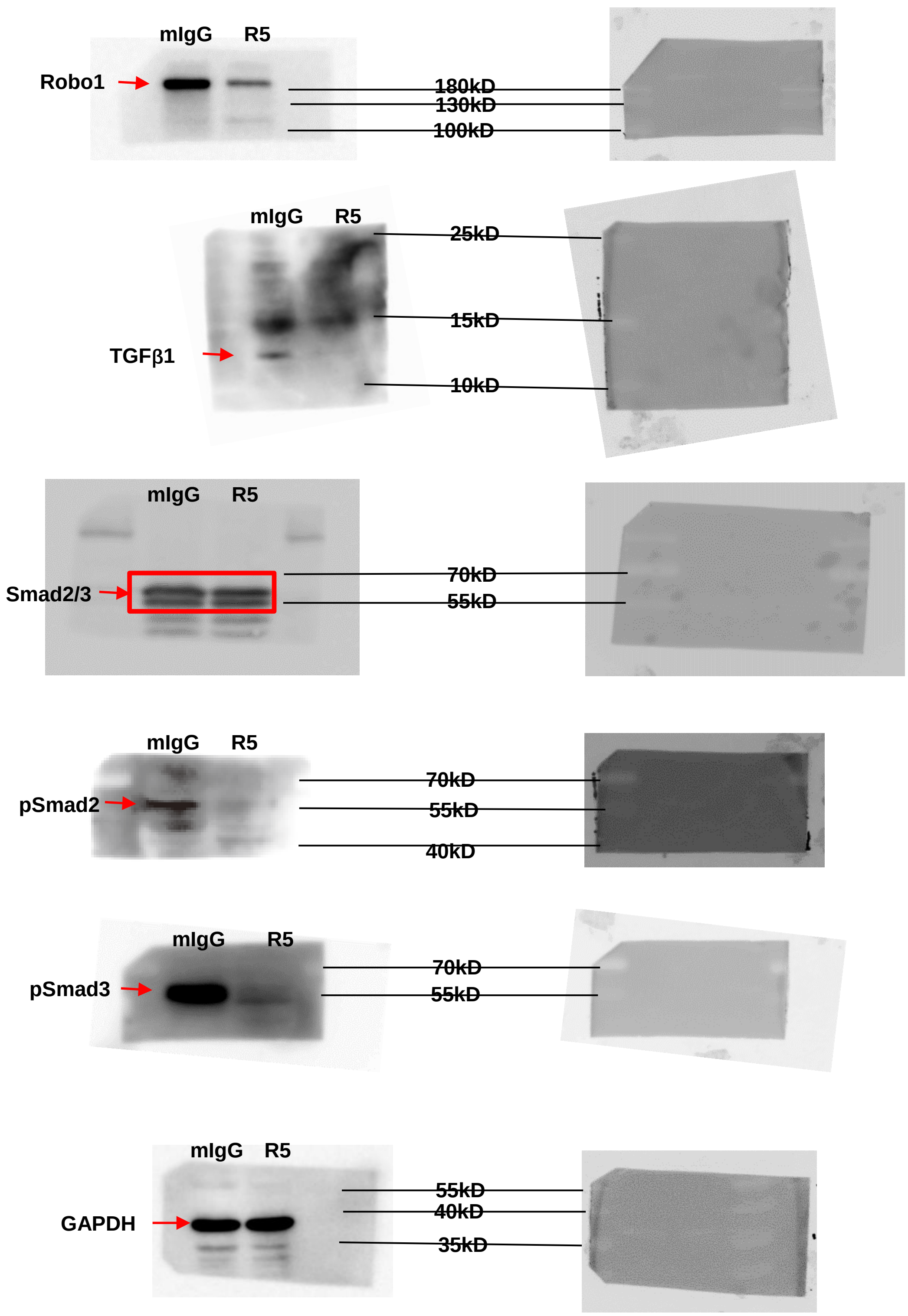
mIgG
R5
Robo1
180kD
130kD
100kD
mIgG
R5
25kD
15kD
TGFβ1
10kD
mIgG
R5
Smad2/3
70kD
55kD
mIgG
R5
pSmad2
70kD
55kD
40kD
mIgG
R5
pSmad3
70kD
55kD
mIgG
R5
GAPDH
55kD
40kD
35kD

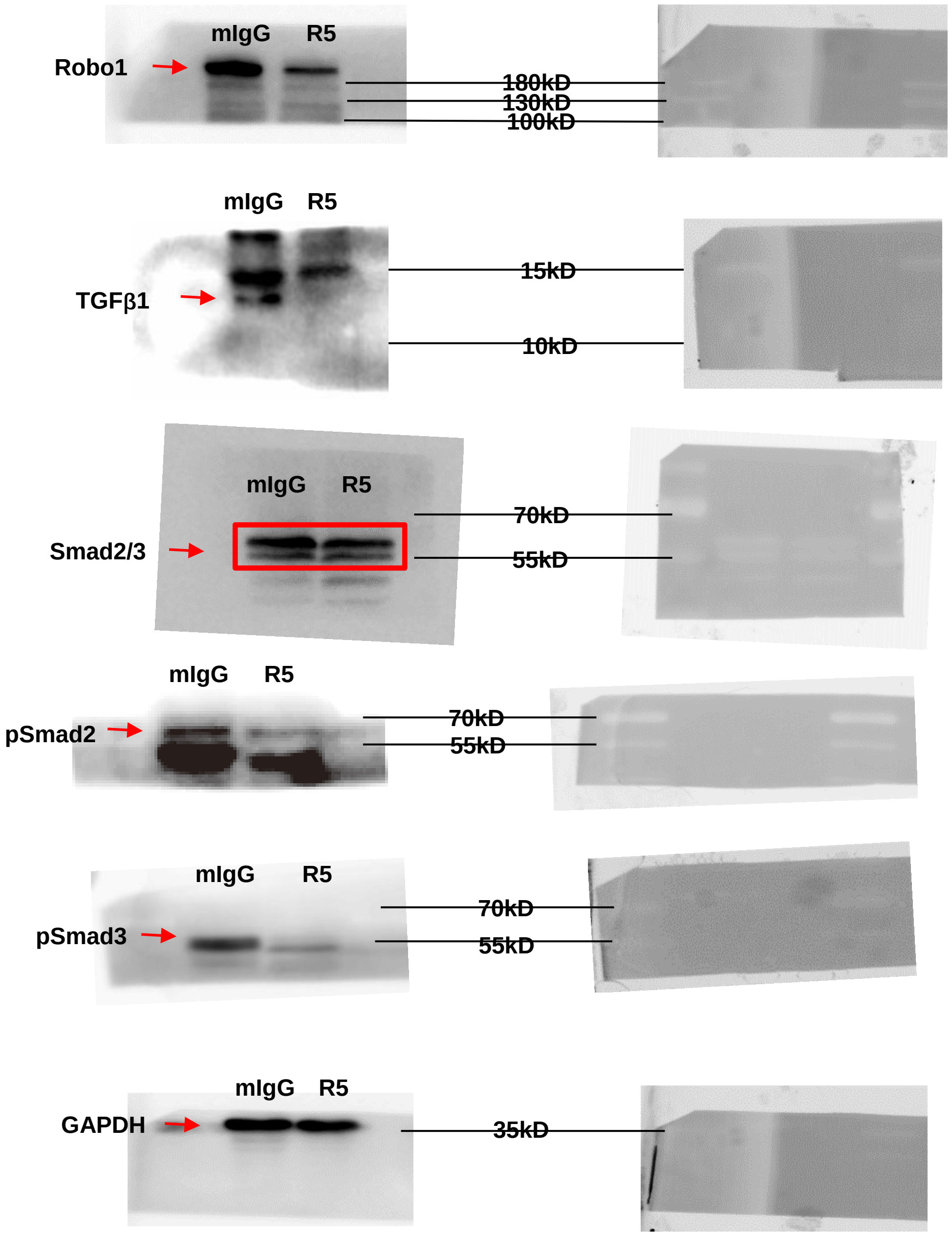
Robo1
mIgG
R5
180kD
130kD
100kD
TGFβ1
mIgG
R5
15kD
10kD
Smad2/3
mIgG
R5
70kD
55kD
pSmad2
mIgG
R5
70kD
55kD
pSmad3
mIgG
R5
70kD
55kD
GAPDH
mIgG
R5
35kD

B. The uncut gels of western blot for three replicates in Supplementary figure 3A.

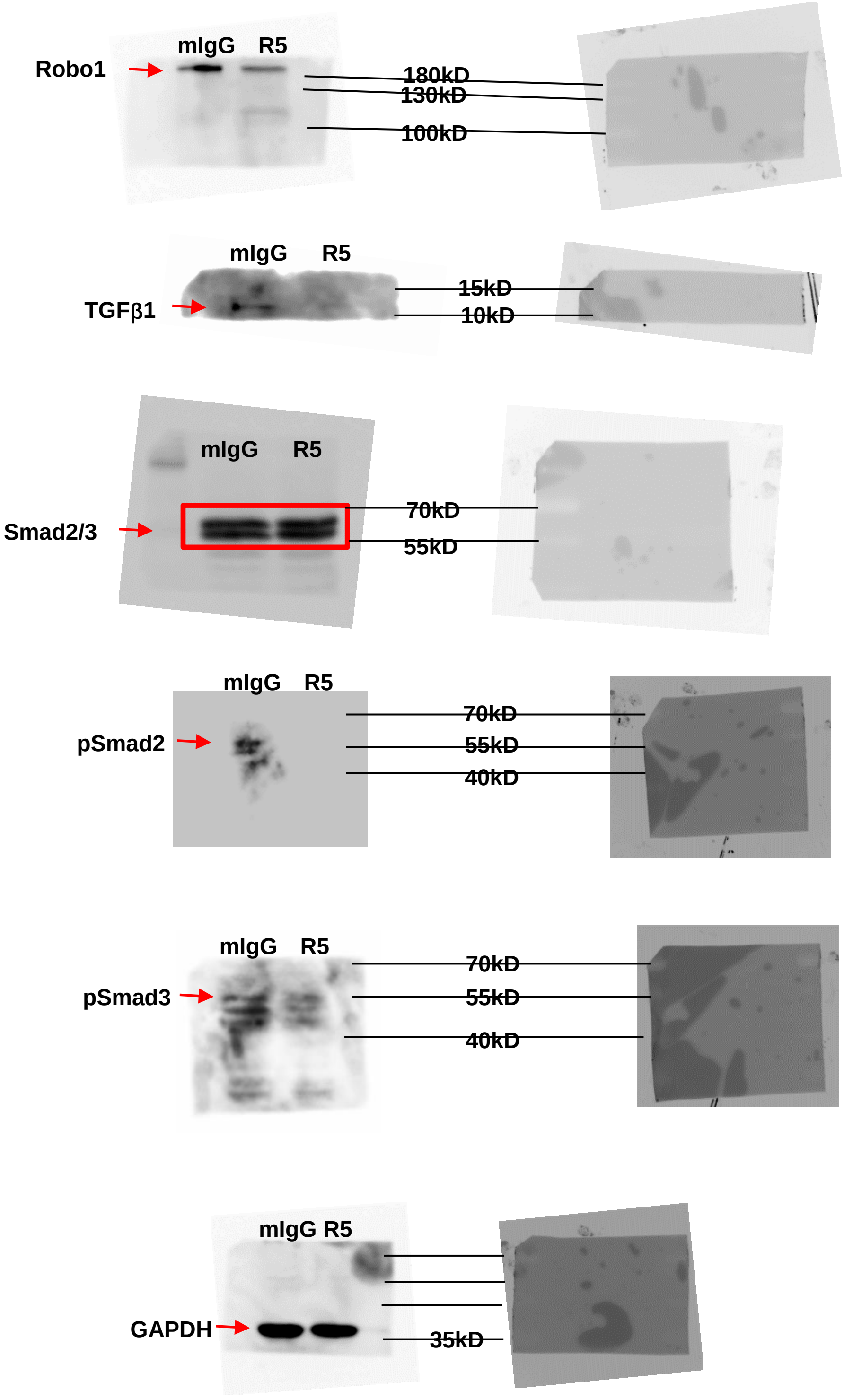

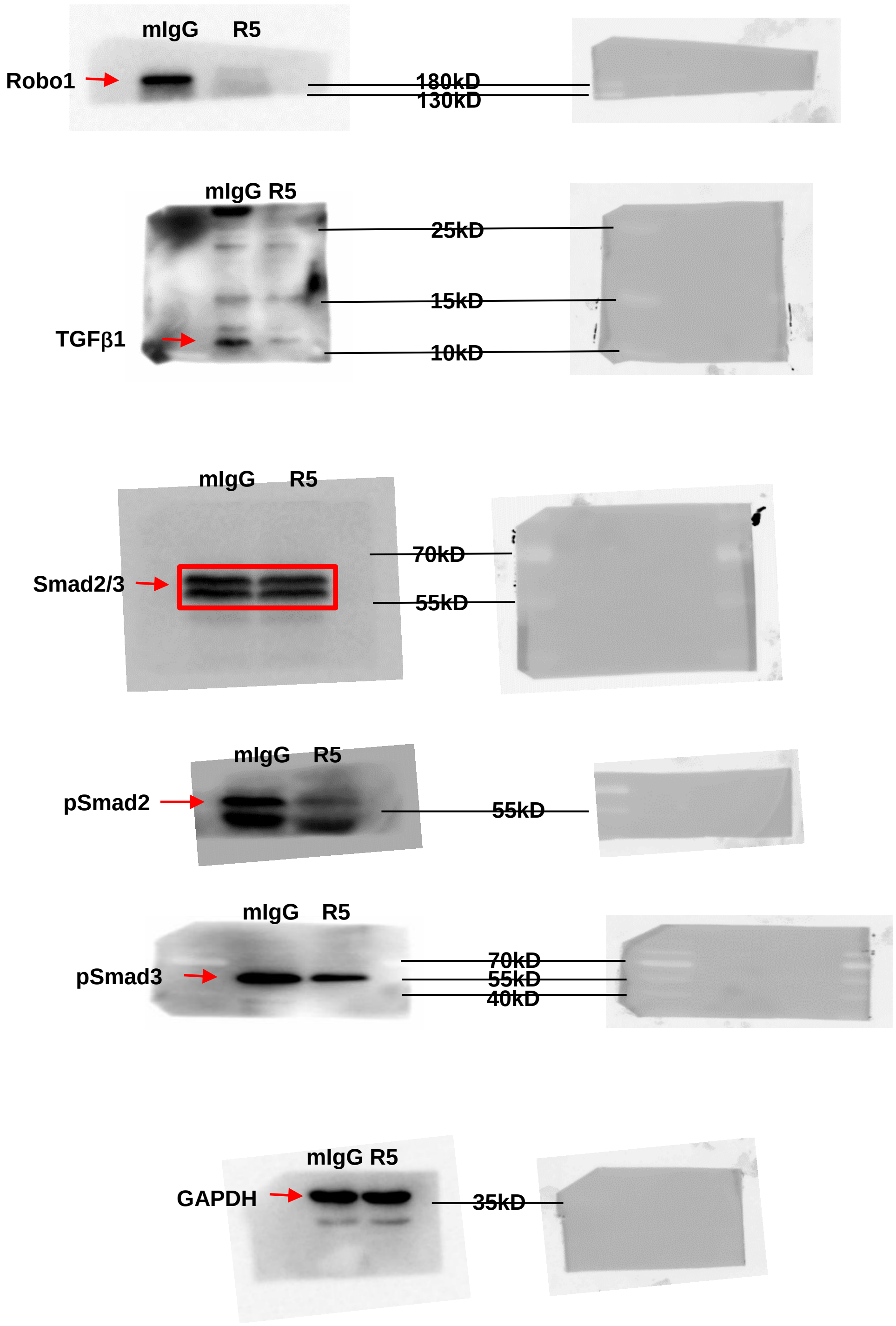
mIgG R5
Robo1
180kD
130kD
mIgG R5
TGFβ1
25kD
15kD
10kD
mIgG R5
Smad2/3
70kD
55kD
mIgG R5
pSmad2
55kD
mIgG R5
pSmad3
70kD
55kD
40kD
mIgG R5
GAPDH
35kD

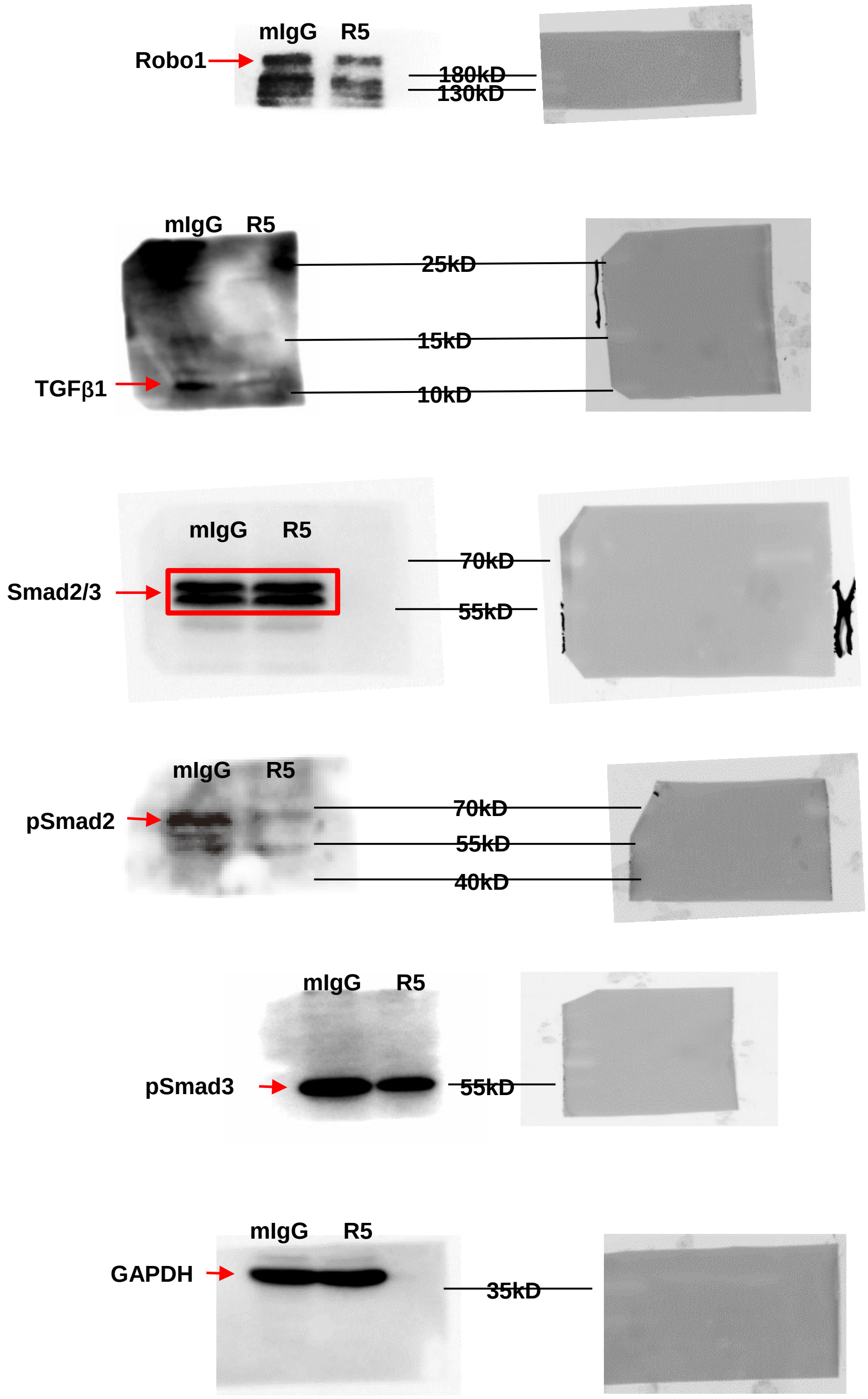

mIgG R5
Robo1
180kD
130kD
mIgG R5
25kD
15kD
TGFβ1
10kD
mIgG R5
70kD
Smad2/3
55kD
mIgG R5
pSmad2
70kD
55kD
40kD
mIgG R5
pSmad3
55kD
mIgG R5
GAPDH
35kD

C. The uncut gels of western blot for two replicates in figure 5D.

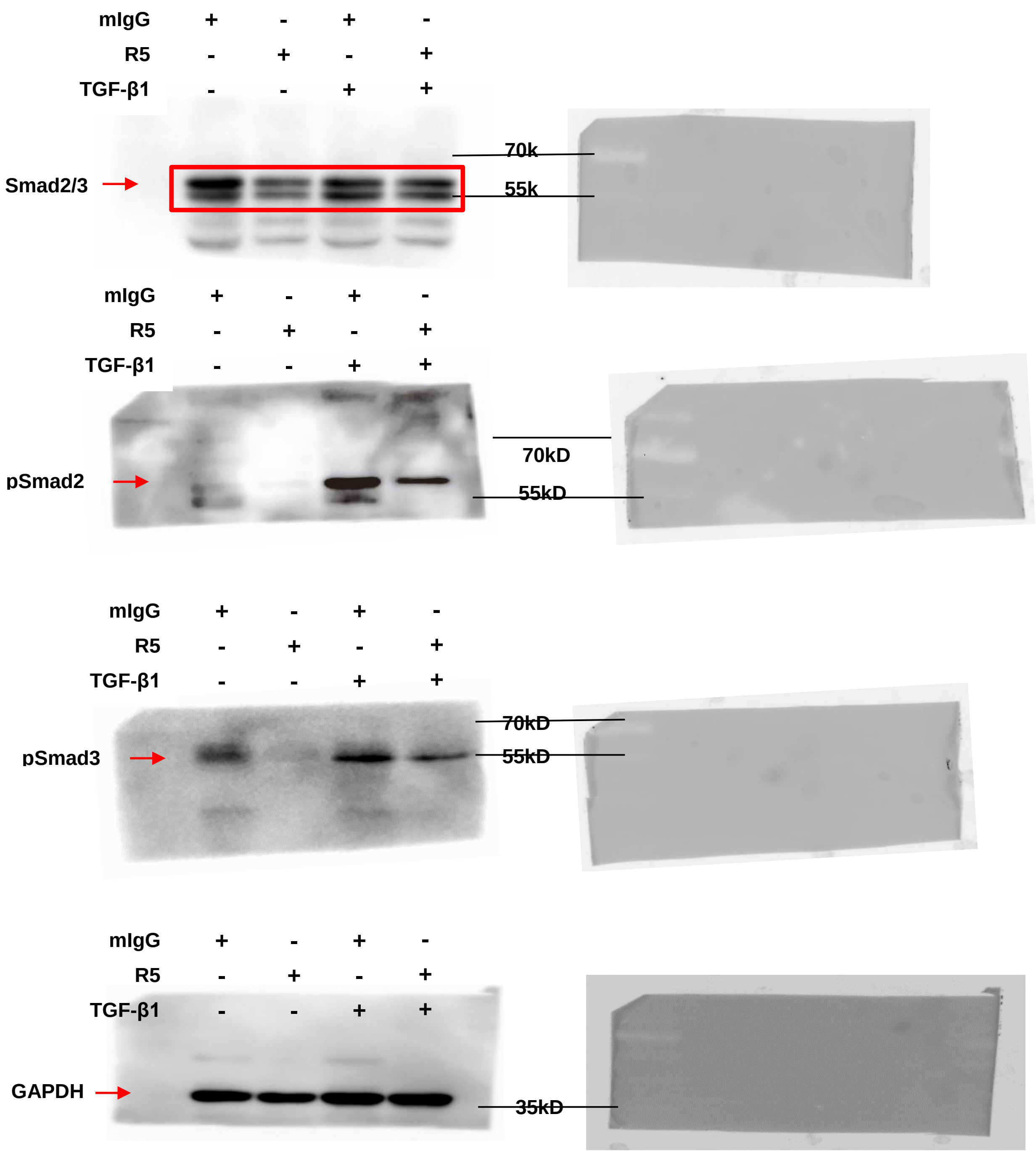

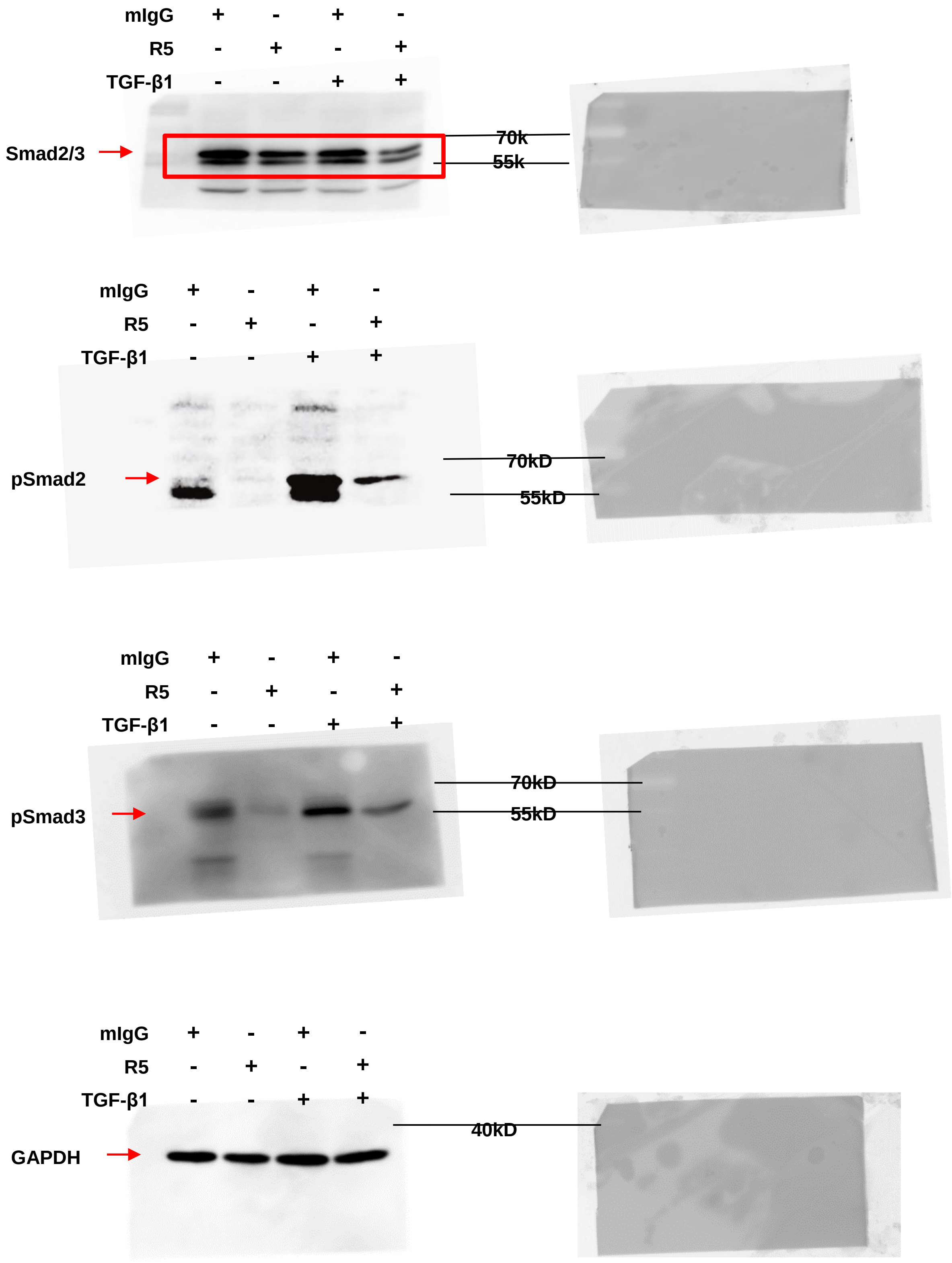
mIgG + - + -
R5 - + - +
TGF-β1 - - + +
Smad2/3
70k
55k
mIgG + - + -
R5 - + - +
TGF-β1 - - + +
pSmad2
70kD
55kD
mIgG + - + -
R5 - + - +
TGF-β1 - - + +
pSmad3
70kD
55kD
mIgG + - + -
R5 - + - +
TGF-β1 - - + +
GAPDH
40kD

Supplementary Table 1. The data of tumor number and tumor burden of $Apc^{Min/+}$ mice that treated with mIgG or R5.

Mouse number	Tumor number						Tumor burden					
	microadenoma		adenoma		total		microadenoma		adenoma		total	
	mIgG	R5	mIgG	R5	mIgG	R5	mIgG	R5	mIgG	R5	mIgG	R5
1	24	13	2	1	26	14	35.467880	11.616490	29.121180	8.588305	64.589060	20.204800
2	25	15	3	1	28	16	34.839840	20.490440	26.554390	9.849745	61.394230	30.340180
3	25	9	3	1	28	10	30.846410	7.981195	36.244480	14.219480	67.090900	22.200680
4	28	15	4	3	32	18	34.017850	15.428870	34.985680	24.309180	69.003520	39.738050
5	27	27	2	3	29	30	28.956180	18.373220	17.166700	49.572060	46.122880	67.945280
